# Sex differences in dynamic cerebral autoregulation responses to resistance and endurance training in humans

**DOI:** 10.1113/EP093183

**Published:** 2026-02-18

**Authors:** Hannah J. Thomas, Channa E. Marsh, Leanne Lester, Louise H. Naylor, Kurt J. Smith, Daniel J. Green

**Affiliations:** ^1^ School of Human Sciences, Exercise and Sport Science The University of Western Australia Perth Western Australia Australia; ^2^ Institute for Health and Sport Victoria University Footscray Victoria Australia; ^3^ Cerebrovascular Health, Exercise, and Environmental Research Sciences Laboratory, Exercise Science, Physical & Health Education University of Victoria Victoria British Columbia Canada

**Keywords:** cerebral blood flow, dynamic cerebral autoregulation, endurance training, resistance training, sex difference

## Abstract

Exercise maintains brain health and reduces the risk of cerebrovascular diseases, such as stroke and dementia. The benefits of different ‘modalities’ of exercise on male and female cerebral autoregulation are unclear. In this study, we compared adaptations in dynamic cerebral autoregulation (dCA) during spontaneous and forced oscillations in blood pressure between males and females following endurance (END) and resistance (RES) training. Using a randomized cross‐over design, 49 healthy adults were recruited to participate in 3 months of END and RES training, with 3 months of washout. Middle cerebral artery blood velocity (MCAv) was measured using transcranial Doppler. The dCA was assessed using spontaneous (at rest) and forced oscillations in blood pressure (squat–stands) in the low‐frequency band (0.167 and 0.083 Hz). At baseline, females had lower end‐tidal partial pressure of CO_2_, higher MCAv and higher gain than males (all *P *< 0.01). On a group level, there was a significant reduction in gain following RES but not END training during spontaneous dCA and forced dCA (all *P *< 0.05). When sexes were analysed separately, this reduction was apparent in females, but not in males. Furthermore, females had a significant reduction in normalized gain during spontaneous dCA and forced dCA at 0.167 Hz. This study is the first to assess sex differences in dCA using a randomized cross‐over design involving supervised exercise of two distinct modalities. We found that RES training might be more beneficial than END training for improving dCA in young healthy individuals, and this is apparent across multiple methods of measuring dCA. This benefit is more apparent in females.

## Abstract (highlights)

- **What is the central question of this study?**
  In this study, we investigated whether different modes of supervised exercise training enhance dynamic cerebral autoregulation in both males and females and whether this would be consistent across multiple methods of measurement.
- **What is the main finding and its importance?**
  We found that resistance training, but not endurance training, might have beneficial impacts on dynamic cerebral autoregulation in young, healthy adults. Furthermore, the benefits of exercise on dynamic cerebral autoregulation might be greater in females than in males.

## 1 INTRODUCTION

The human brain requires a high proportion of resting oxygen uptake and possesses a limited oxygen reserve (Willie et al., 2014). Coupled with the bony skull surrounding the brain allowing minimal tissue expansion, it is critical to regulate cerebral blood flow (CBF) tightly. One of the key mechanisms facilitating constant CBF to the brain is cerebral autoregulation (CA) (Willie et al., 2014). CA can be defined as the intrinsic ability of the brain to regulate CBF independently, via changes in cerebrovascular resistance, in response to hypo‐ and hypertensive blood pressure fluctuation. Specifically, dynamic CA (dCA) refers to CBF responses to transient fluctuations in arterial blood pressure. However, the quantification of dCA remains complex and poorly understood owing to the lack of a gold‐standard examination.

In a recent article, Brassard et al. (2024) addressed the challenges of assessing dCA. The authors highlighted that, over the years, investigators have applied numerous assessment and analytical methods and approaches to quantify dCA based on either a single rapid change or oscillations (spontaneous or forced) in arterial blood pressure (Brassard et al., 2023). Spontaneous assessments are advantageous for clinical populations, but forced oscillations in blood pressure and cerebral blood velocity result in more robust coherence values to enhance the interpretation of the linear association between blood pressure and cerebral blood velocity (Burma et al., 2024; Smirl et al., 2015). Furthermore, differences in the stressor (magnitude and duration of blood pressure fluctuations) used can result in distinctive differences in the regulatory mechanisms activated during dCA. Few studies investigating exercise training have used a multi‐method strategy (both spontaneous and forced) when assessing dCA.

Exercise has complex impacts on the cardiovascular system, and the modality of exercise [endurance (END) vs. resistance (RES)] has impacts on regulation and adaptation, including that of brain perfusion (Ainslie et al., 2008). Habitual END training has been shown to reduce the age‐related decline in cerebral perfusion (Ainslie et al., 2008), and supervised RES exercise training has recently been shown to increase cerebrovascular reactivity to carbon dioxide (Thomas et al., 2021b, 2022). The chronic effect of RES and END exercise on cerebrovascular capacity to respond to rapid changes in blood pressure (i.e., dCA) has been examined previously, with inconsistent results. Some research has suggested that life‐long endurance training might result in less effective dCA in both young healthy adults and masters athletes (Aengevaeren et al., 2013; Lind‐Holst et al., 2011), whereas others have reported that there is no detrimental impact of endurance training, with no differences between endurance‐trained and untrained individuals (Ichikawa et al., 2013). Furthermore, some studies have also shown that dCA is unaltered in RES‐trained individuals (Perry et al., 2019; Roy et al., 2022); however, research on the impacts of RES training on dCA is lacking. This variability in the impact of exercise on dCA across the literature is probably, in part, a consequence of the inconsistent methodological approaches in the assessment of autoregulatory capacity (Smirl et al., 2015). However, of particular importance is that previous research has relied primarily on cross‐sectional comparisons of dCA between habitual exercisers and sedentary control subjects. A more robust experimental design would involve supervised longitudinal exercise‐training interventions to characterize the benefits of different exercise modalities on dCA within subjects, with multiple metrics of dCA (spontaneous and forced).

In a recent study, we used a randomized cross‐over experimental design involving 12 week supervised exercise interventions of both RES and END training in the same individuals, and we reported no significant benefits of training on dCA (Thomas et al., 2021b). However, in that paper we presented only spontaneous dCA data, meaning we might have overlooked changes that could have been seen using forced dCA owing to the increased signal‐to‐noise ratio of this technique. This is especially true considering that the use of transfer function analysis for data interpretation does not incorporate the directional sensitivity of the cerebral pressure–flow relationship. Furthermore, it is possible that impairment or changes in dCA might result in slowing of the adaptative response to training, which might occur earlier with rapid blood pressure changes (forced dCA) in comparison to slower changes in blood pressure (spontaneous dCA) (Claassen et al., 2021). In the present paper, we present data from both the spontaneous and forced dCA of individuals who underwent both RES and END, to understand whether there are differences in the rate of adaptation.

Despite a recent push in academia to study sex as a biological variable, there remains a lack of information on the differences between males and females, specifically relating to complex physiological adaptations to exercise interventions. For example, the prevalence of cerebrovascular disease, such as stroke, is lower in young women compared with men; however, stroke rates increase disproportionally in women around midlife such that rates become similar and eventually surpass those of men (Benjamin et al., 2018). In young individuals, there are mixed results regarding sex differences in cerebral autoregulation. Labrecque et al. (2019) recently reported that the cerebrovasculature of fit women has an attenuated ability to react to rapid changes in blood pressure in the face of preserved orthostasis, after comparing 11 fit women to 11 age‐matched men. Favre and Serrador (2019) investigated the sex differences in dCA, in addition to the impact of female sex hormones. They reported no difference in dCA across the menstrual cycle in females but found significantly improved dCA in the middle cerebral artery (MCA) of females compared with males. Despite these significant findings and the continued lack of clarity, sex differences in responses to distinct modes of exercise training are yet to be investigated comprehensively.

In this study, we aimed to compare differences in dCA responses between males and females following a supervised intervention of END and RES training. We hypothesized that changes in dCA between males and females would be similar and that RES and END training would have similar impacts on dCA regulation. Furthermore, we aimed to compare dCA as measured during both spontaneous and forced blood pressure oscillations. We hypothesized that forced dCA would reveal greater changes with training than spontaneous dCA.

## 2 MATERIALS AND METHODS

Full details of the study design and experimental procedures can be found in our protocol paper (Marsh et al., 2020) and in the study registration (ACTRN12616001095459), which was published prior to recruitment and randomization. A summary is provided below. Of note, this study was conducted on pairs of monozygotic and dizygotic twins. However, for the purpose of this report, we present sex difference responses only, and heritability analyses are not included. Nonetheless, we have adjusted all analyses for twin correlations. Analysis comparing group and twin responses to exercise training for spontaneous dCA in a larger sample can be found published elsewhere (Thomas et al., 2021b, 2022).

### 2.1 Participants

Forty‐nine young, healthy adults (25 ± 5 years; 15 males and 34 females) completed the cerebrovascular component of the study. Participants were asked to relate their sex assigned at birth and to describe their gender. All individuals reported their gender as consistent with their sex recorded at birth. Recruitment was facilitated via newsletters, mail, newspaper advertisements, online and via social media, university email lists and word‐of‐mouth referrals. Inclusion criteria were healthy, relatively sedentary individuals who did not meet Australian guidelines for physical activity recommendations (<150 min per week), were non‐smokers and medication free. Individuals with a history of previous RES or END training were not excluded. This study was approved by the University of Western Australia Human Research Ethics Committee (reference number RA/4/7031). Oral and written consent were obtained from all subjects prior to participation in the study, which conformed to the *Declaration of Helsinki*.

### 2.2 Study design

Cerebral autoregulation measures were undertaken by all participants within 14 days of commencing and completing each intervention, as detailed below. Participants were randomized to 3 months of either RES or END exercise training, before undergoing a 3 month washout period, during which they were instructed to return to their usual activities and diet, which was assessed using food diaries and activity monitors (Thomas et al., 2021a). Participants then crossed over to complete 3 months of their second, alternative, exercise intervention (RES or END). Randomization was performed using a website‐based randomization tool after baseline testing was completed.

### 2.3 Exercise interventions

The centre‐based, supervised exercise interventions consisted of three 1 h sessions per week for 12 weeks. The programmes were intensity [heart rate (HR)] matched between volunteers and progressively overloaded across the 12 weeks, consisting of specified training phases. The exercise modalities were not matched for workload or energy expenditure; we opted to assess the impact of ecologically valid exercise prescriptions typical of those used in gymnasia and industry settings.

END used two running and one cycling session per week. Duration and intensity increased progressively over the course of the 3 month exercise intervention from 15 to 60 min of exercise and from 60% to 90% peak oxygen consumption, which was monitored via continuous HR, allowing the trainer to ensure that participants were working at their specified HRs for each session (Polar RS300X HR monitor, Polar Electro Oy, Finland). Target HRs were calculated from HR at a percentage of the initial graded exercise peak oxygen consumption test, hence they were individualized, but matched for intensity between individuals. The 12‐week END programme followed a periodized progressive macrocycle plan consisting of three mesocycles of 4 weeks each. The first mesocycle, the ‘general preparatory’ phase (weeks 1–4), consisted of low training intensity/volume (walking/jogging at 60% HR, 1–2.5 km running and/or 15–25 min cycling) including a long warm‐up focusing on running drills, technique and dynamic stretching. The second mesocycle, the ‘high‐intensity’ phase (weeks 5–8), consisted of higher intensity work with higher HRs (≤90%) and distance/duration (2.5–5 km running and/or 25–40 min cycling). The final mesocycle, the ‘maintenance and distance’ phase (weeks 9–12), consisted mostly of maintaining subthreshold HRs (70%–85%) for longer distance/duration (from 5–7 km running and/or 40 min in week 9 to 60 min cycling by week 12). In the mesocycles, a 1:1 structure loading existed, with a ‘hard’ loading week followed by an ‘easier’ or ‘maintenance’ week. Within each week, there existed a structured load with a ‘harder’ run session, an ‘easier’ run session, and the cycling session was always the longest duration session in each week (Daniels, 2013; Marsh et al., 2020; Spence et al., 2011).

RES alternated between upper‐ and lower‐body exercises and sessions that progressed from 60% to 90% of one‐repetition maximum (1RM). RES was monitored by recording the number of repetitions, sets and weight completed, in addition to a rating of perceived exertion for each set. Participants were instructed and coached to avoid the Valsalva manoeuvre to prevent exacerbations in blood pressure during exercise and subsequent postexercise hypotension. Individual weights were prescribed from each participant's pretraining 1RM, hence they were individualized, but matched for relative intensity between individuals. Specific exercises, number of sets and number of repetitions were standardized across all participants. Each session focused on one of the five main exercises [two upper body (bench press and standing military press) and three lower body (squats, deadlift and leg press)], alternating upper and lower body on separate days. There were secondary exercises performed during each session that used other muscle groups (i.e., staggered feet leg press, seated row and lat pulldown). Participants performed a standardized warm‐up before completing their session and a standardized 5 min of core exercises and cool‐down at the end of the session. To guide participants’ progressions, 1RM assessments were repeated halfway through their 12 week programme (Marsh et al., 2020). For specific detail about the mesocycle training phases, including the intensity, number of repetitions and rest periods, please refer to the published protocol paper (Marsh et al., 2020).

### 2.4 Outcome measures

Participants arrived at the laboratory at the same time each morning for repeated measures following an overnight fast. Participants had refrained from any moderate/vigorous physical activity and alcohol for 24 h and caffeine for 12 h before the testing session. This was confirmed at the start of each session verbally and recorded on the testing sheet. Participants’ menstrual cycle phase and use of contraception were recorded prior to each testing session. Owing to the nature of this intervention (i.e., having multiple data‐collection days at strict time points and twins in a pair having to train at the same time), it was not possible to control for menstrual cycle phase.

Middle cerebral artery velocity (MCAv) was measured using non‐invasive transcranial Doppler (TCD, Spencer Technologies, Seattle, WA, USA), according to standardized approaches (Willie et al., 2011). Vessels were measured at a frequency of 100 Hz, at recommended depths, described in TCD guidelines (Willie et al., 2011) (depth varied between participants). To standardize vessel recording sites within participants, photographs were taken of the probe position, TCD settings and velocity traces, which were used in follow‐up testing sessions. The coefficient of variation for repeated assessment of resting MCAv is 4.5% (Ainslie et al., 2008) using TCD.

A Finometer (Finometer Pro, Finapres Medical Systems, Amsterdam ZO, The Netherlands) continuously monitored beat‐to‐beat blood pressure via finger arterial volume clamping from the middle finger of the left hand. A three‐lead ECG was attached to monitor HR (BIO Amp CF, ADInstruments, NSW, Australia). Following instrumentation, the procedure was explained to participants in detail, a mouthpiece connected to a spirometer (Spirometer, ADInstruments, New South Wales, Australia) was placed in the participant's mouth, with a nose clip, to measure expired gases. Breath‐by‐breath CO_2_ and O_2_ were sampled at the mouth and recorded using a calibrated gas analyser (ML206, ADInstruments, CO, USA). The partial pressures of end‐tidal CO_2_ ($P_{\mathrm{ET},CO_{2}}$) and O_2_ were calculated in LabChart using peak detection analysis, with correction for daily barometric pressure. The blood pressure, ECG and respiratory signals were recorded on LabChart 8 via a 16‐channel Powerlab (ADinstruments, Sydney, NSW, Australia). The cerebrovascular resistance (MCA resistance) was calculated as mean arterial pressure (MAP) divided by MCAv (in millimetres of mercury per centimetre per second). The MCA pulsatility index (MCA PI) was estimated as systolic MCAv minus diastolic MCAv divided by mean MCAv (De Riva et al., 2012).

Once instrumented, the participant was seated on a semi‐supine bench (the incline of the bench remained consistent to allow optimal reproducibility of blood vessel images), and a 5 min resting baseline of the above cerebrovascular and cardiorespiratory parameters was recorded. dCA was then assessed during forced oscillations in arterial blood pressure, induced by repeated squat–stand manoeuvres, at two speeds (0.167 and 0.083 Hz) in the low‐frequency range.

#### 2.4.1 Dynamic cerebral autoregulation

dCA was assessed using transfer function analysis of both spontaneous and forced oscillations in blood pressure versus MCAv. Data were processed and analysed using the commercially available software Ensemble (v.1.0.0.42, Elucimed Ltd, University of Otago, New Zealand) in accordance with the recommendations of the Cerebral Autoregulation Research Network (CARNet) white paper on transfer function analysis (Claassen et al., 2016). Transfer function analysis coherence, phase, gain and normalized gain (ngain) values were sampled at two‐point estimates within the low‐frequency (0.167 and 0.083 Hz) domain, based on methodological recommendations (Green et al., 2018). Coherence refers to the linearity of the relationship between the input (blood pressure) and output (cerebral blood velocity) (fraction of blood pressure that is linearly related to MCAv), which ranges from 0.0 (no relationship) to 1.0 (completely linear relationship) (Zhang et al., 1998). Phase describes the timing buffer between the input and output variables (radians and degrees; difference of the timing of the blood pressure and MCAv waveforms). Gain refers to the amplitude modulation within the cerebral pressure–flow relationship (amplitude of MCAv change for a given oscillation in MAP), and ngain is the product of substituting gain by the product of gain and cerebrovascular resistance (as a percentage per millimetre of mercury), enabling a comparison between the various interventions in the study that resulted in alterations to either blood pressure or cerebral blood velocity (in centimetres per second per millimetre of mercury) (Van Beek et al., 2008; Zhang et al., 1998).

Spontaneous dCA was calculated from a 5 min period of rest, and the forced oscillations were induced via repeated squat–stands at 0.083 Hz (12 s cycle: 6 s squat–6 s standing) and 0.167 Hz (6 s cycle: 3 s squat–3 s standing), timed using a metronome. For each frequency, participants stood for 1 min prior to commencing 3 min of squat–stand manoeuvres, separated by 5 min of seated recovery to ensure that all cardiovascular variables returned to baseline. Both squatting cycle lengths represent a frequency within the low‐frequency range (0.07–0.20 Hz) (Claassen et al., 2016), which is within the range where dCA is thought to have its greatest influence on cerebral pressure–flow dynamics (Zhang et al., 1998). To complete the squat–stand manoeuvres, participants began in a standing position and proceeded to squat down until their fingers reached the top of their knees, which was demonstrated first by the investigator. This squat position was held for a set duration before returning to the standing position. The study protocol was finalized prior to publication of the current CARNet guidelines and, as such, varies slightly from the currently accepted methods (e.g., 3 min was used, in comparison to the current recommendation of 5 min). The chosen frequency of manoeuvres (0.167 and 0.083 Hz) was used to replicate the number of squat–stand cycles that would be performed in the more traditional (0.1 Hz, 30 cycles and 0.05 Hz, 15 cycles) frequencies when using 3 compared with 5 min.

To evaluate the change in $P_{\mathrm{ET},CO_{2}}$ and blood pressure for each squatting frequency, the first and last five breaths were averaged. The difference between these two values represents the change in $P_{\mathrm{ET},CO_{2}}$ and blood pressure at that frequency. These change scores were assessed to ensure that there was no change in $P_{\mathrm{ET},CO_{2}}$ or blood pressure across the dCA task, subsequently affecting the MCAv results. Furthermore, these changes were compared across dCA assessments and across time points.

Where values were identified as wrap‐around phase, data were excluded from analysis (Claassen et al., 2016). Critical values of coherence were calculated based on the CARNet guidelines. Five windows were adopted for data segmentation using Welch's method at the 95% significance level, meaning that all coherence values <0.34 were excluded from analysis, to ensure measures were robust for subsequent analysis (Panerai et al., 2023). These methodological approaches are in accordance with published guidelines on this technique (Meel‐Van Den Abeelen et al., 2014). Data values outside 3SD were also removed from analysis. Participant full data sets were excluded if they did not have data for all four time points (RES and END). After all exclusions, 49 participants were included for spontaneous dCA analysis: 36 for forced dCA at 0.083 Hz and 49 for forced dCA at 0.167 Hz. The lower number of participants included for the forced dCA at 0.083 Hz was predominantly attributable to data quality issues.

### 2.5 Statistical analysis

Statistical analyses were performed with STATA SE17 software (StataCorp, College Station, TX, USA). Differences between sexes (Table 1) and the effect of the exercise interventions on each outcome measure (Tables 2 and 3) were assessed using a linear mixed model, which accounted for the repeated nature of the data, controlling for individual fixed effects, which considers the twin association. The fixed effects of the linear mixed model were specified as regression parameters (time, workload and age), and the random effects portion of the model were specified by considering the grouping structure of the data (twin pairings). The time point and participant random effects were structured as separate nested measurements. Comparisons of $P_{\mathrm{ET},CO_{2}}$ and blood pressure at the beginning compared with the end of each dCA bout and the comparisons of $P_{\mathrm{ET},CO_{2}}$ across time points were analysed using a mixed effects regression model (assessed for a subset of 22 and 21 participants, respectively, who had all data for all conditions). The α confidence value for statistical significance was defined as *P* < 0.05. All data are presented as the mean (SD).

**TABLE 1:** Female and male baseline characteristics.

Characteristic	Female	Male	*P*‐value
Baseline	*n* = 34	*n* = 15	
Age, years	24 (5)	27 (5)	0.186
MAP, mmHg	91 (11)	87 (10)	0.163
$P_{\mathrm{ET},CO_{2}}$, mmHg	38.7 (2.6)	42.2 (2.0)	**0.010**
MCAv mean, cm s^−1^	78.2 (14.9)	66.4 (14.1)	**0.002**
MCA resistance, mmHg cm^−1^ s^−1^	1.231 (0.387)	1.354 (0.280)	0.117
MCA PI	0.770 (0.109)	0.744 (0.082)	0.517
HR, beats min^−1^	76 (8)	71 (10)	0.205
Coherence	0.81 (0.09)	0.85 (0.06)	0.871
Gain, cm s^−1^ mmHg^−1^	1.05 (0.27)	0.80 (0.13)	**0.000**
ngain, % mmHg^−1^	1.36 (0.33)	1.24 (0.24)	0.091
Phase, rad	0.41 (0.17)	0.44 (0.18)	0.424

*Note*: Transfer function analysis metrics are obtained from the spontaneous dynamic cerebral autoregulation data. Data are the mean (SD) Abbreviations: HR, heart rate; MAP, mean arterial pressure; MCAv, middle cerebral artery velocity; $P_{\mathrm{ET},CO_{2}}$, partial pressure of end‐tidal carbon dioxide; PI, pulsatility index. Values in bold are statistically significant (*P* < 0.05).

**TABLE 2:** Group mean changes with resistance and endurance training.

	Baseline (RES)	∆RES	*P*‐value	Baseline (END)	∆END	*P*‐value	*P*‐value (∆RES vs. ∆END)
Low‐frequency BL (*n* = 49)							
Coherence	0.79 (0.12)	0.01 (0.14)	0.558	0.82 (0.09)	−0.05 (0.12)	**0.003**	0.137
Gain	0.97 (0.25)	−0.08 (0.22)	**0.009**	0.97 (0.28)	−0.05 (0.24)	0.175	0.440
ngain	1.31 (0.31)	−0.07 (0.30)	0.098	1.32 (0.29)	−0.04 (0.31)	0.369	0.596
Phase	0.47 (0.20)	−0.02 (0.21)	0.479	0.43 (0.16)	0.02 (0.17)	0.423	0.248
Low frequency 0.167 Hz (*n* = 36)							
Coherence	0.84 (0.09)	0.00 (0.11)	0.954	0.81 (0.10)	0.04 (0.11)	0.056	0.114
Gain	0.81 (0.22)	−0.11 (0.21)	**0.002**	0.70 (0.22)	0.05 (0.30)	0.330	**0.009**
ngain	1.21 (0.32)	−0.06 (0.31)	0.254	1.04 (0.27)	0.11 (0.40)	0.094	**0.043**
Phase	0.69 (0.27)	0.05 (0.39)	0.400	0.64 (0.41)	−0.02 (0.49)	0.808	0.476
Low frequency 0.083 Hz (*n* = 49)							
Coherence	0.98 (0.02)	0.01 (0.03)	0.103	0.98 (0.02)	0.00 (0.03)	0.872	0.229
Gain	0.83 (0.18)	−0.06 (0.22)	**0.047**	0.81 (0.21)	−0.04 (0.23)	0.224	0.596
ngain	1.28 (0.20)	−0.01 (0.19)	0.813	1.27 (0.27)	−0.02 (0.33)	0.680	0.812
Phase	0.45 (0.19)	−0.01 (0.17)	0.610	0.45 (0.17)	−0.01 (0.13)	0.450	0.961

*Note*: Completers analysis. Data are mean (SD). Abbreviations: BL, spontaneous dynamic cerebral autoregulation; END, endurance training; RES, resistance training. Values in bold are statistically significant (*P* < 0.05).

**TABLE 3:** Female and male baseline values and group mean changes with training.

	Baseline		△ Exercise				
**Resistance**	**F (*n* = 34)**	**M (*n* = 15)**	**F (*n* = 34)**	***P*‐value**	**M (*n* = 15)**	***P*‐value**	**F vs. M (*P*‐value)**
Coherence (LF BL)	0.78 (0.12)	0.79 (0.14)	−0.04 (0.13)	0.079	0.05 (0.14)	0.137	**0.026**
Gain (LF BL)	1.04 (0.26)	0.82 (0.16)	−0.10 (0.25)	**0.022**	−0.05 (0.13)	0.160	0.449
ngain (LF BL)	1.35 (0.33)	1.23 (0.26)	−0.11 (0.31)	**0.036**	−0.02 (0.26)	0.746	0.145
Phase (LF BL)	0.46 (0.19)	0.48 (0.21)	0.00 (0.22)	0.907	−0.06 (0.16)	0.156	0.399
**Resistance**	**F (*n* = 24)**	**M (*n* = 12)**	**F (*n* = 24)**	***P*‐value**	**M (*n* = 12)**	***P*‐value**	**F vs. M (*P*‐value)**
Coherence (LF 0.167 Hz)	0.84 (0.09)	0.85 (0.10)	−0.01 (0.10)	0.502	0.02 (0.13)	0.505	0.326
Gain (LF 0.167 Hz)	0.85 (0.21)	0.73 (0.23)	−0.15 (0.22)	**0.001**	−0.03 (0.17)	0.581	0.086
ngain (LF 0.167 Hz)	1.22 (0.31)	1.19 (0.35)	−0.12 (0.32)	**0.046**	0.07 (0.25)	0.299	0.055
Phase (LF 0.167 Hz)	0.65 (0.23)	0.76 (0.34)	0.08 (0.41)	0.354	0.01 (0.36)	0.921	0.625
**Resistance**	**F (*n* = 33)**	**M (*n* = 16)**	**F (*n* = 33)**	***P*‐value**	**M (*n* = 16)**	***P*‐value**	**F vs. M (*P*‐value)**
Coherence (LF 0.083 Hz)	0.98 (0.02)	0.98 (0.02)	0.01 (0.03)	0.056	0.00 (0.03)	0.860	0.348
Gain (LF 0.083 Hz)	0.87 (0.15)	0.73 (0.20)	−0.08 (0.26)	**0.049**	−0.02 (0.13)	0.496	0.352
ngain (LF 0.083 Hz)	1.30 (0.19)	1.24 (0.22)	−0.02 (0.21)	0.525	0.03 (0.15)	0.424	0.375
Phase (LF 0.083 Hz)	0.42 (0.16)	0.51 (0.22)	−0.02 (0.14)	0.427	0.00 (0.22)	0.965	0.672
**Endurance**	**F (*n* = 34)**	**M (*n* = 15)**	**F (*n* = 34)**	***P*‐value**	**M (*n* = 15)**	***P*‐value**	**F vs. M (*P*‐value)**
Coherence (LF BL)	0.81 (0.08)	0.84 (0.10)	−0.05 (0.12)	**0**.**011**	−0.05 (0.12)	0.100	0.987
Gain (LF BL)	1.06 (0.26)	0.76 (0.21)	−0.07 (0.25)	0.089	0.01 (0.22)	0.827	0.252
ngain (LF BL)	1.39 (0.28)	1.18 (0.27)	−0.09 (0.29)	0.078	0.07 (0.34)	0.429	0.098
Phase (LF BL)	0.43 (0.16)	0.42 (0.16)	0.02 (0.19)	0.541	0.02 (0.15)	0.574	0.980
**Endurance**	**F (*n* = 24)**	**M (*n* = 12)**	**F (*n* = 24)**	***P*‐value**	**M (*n* = 12)**	***P*‐value**	**F vs. M (*P*‐value)**
Coherence (LF 0.167 Hz)	0.80 (0.10)	0.84 (0.11)	0.03 (0.11)	0.262	0.06 (0.11)	0.076	0.437
Gain (LF 0.167 Hz)	0.72 (0.22)	0.66 (0.21)	0.07 (0.33)	0.262	−0.01 (0.2)	0.932	0.441
ngain (LF 0.167 Hz)	1.00 (0.26)	1.12 (0.27)	0.15 (0.45)	0.275	0.04 (0.27)	0.890	0.355
Phase (LF 0.167 Hz)	0.61 (0.40)	0.68 (0.43)	−0.06 (0.51)	0.573	0.06 (0.48)	0.676	0.511
**Endurance**	**F (*n* = 33)**	**M (*n* = 16)**	**F (*n* = 33)**	***P*‐value**	**M (*n* = 16)**	***P*‐value**	**F vs. M (*P*‐value)**
Coherence (LF 0.083 Hz)	0.97 (0.03)	0.99 (0.01)	0.00 (0.03)	0.419	−0.01 (0.02)	**0.003**	0.061
Gain (LF 0.083 Hz)	0.84 (0.20)	0.74 (0.20)	−0.04 (0.24)	0.383	−0.05 (0.21)	0.365	0.873
ngain (LF 0.083 Hz)	1.24 (0.25)	1.31 (0.30)	0.01 (0.32)	0.901	−0.07 (0.36)	0.406	0.421
Phase (LF 0.083 Hz)	0.44 (0.17)	0.47 (0.16)	−0.01 (0.12)	0.774	−0.03 (0.14)	0.395	0.541

Completers analysis. Data are the mean (SD). Abbreviations: BL, spontaneous dynamic cerebral autoregulation; END, endurance training; F, female; LF, low‐frequency domain; M, male; RES, resistance training. Values in bold are statistically significant (*P* < 0.05).

## 3 RESULTS

### 3.1 Participant characteristics

Baseline characteristics for the 49 participants included in this study are provided in Table 1.

### 3.2 Exercise training efficacy

Attendance at training sessions was 94% for RES and 95% for END. As previously published (Marsh et al., 2020), peak oxygen consumption increased significantly by 3.61 ± 3.77 mL kg^−1 ^min^−1^ in response to END (0.25 ± 0.26 L min^−1^, *P *< 0.005), but not in response to RES (0.03 ± 3.57 mL kg^−1^ min^−1^; 0.04 ± 0.25 L min^−1^). In contrast, 1RM increased significantly in response to RES (leg press, +47.0 ± 29.4 kg, *P *< 0.01; bench press, +5.1 ± 5.0 kg, *P *< 0.01) but not END (leg press, +3.0 ± 26.4 kg; bench press, −0.4 ± 3.4 kg). Both RES and END significantly increased total lean mass (1156 ± 1132 g, *P *< 0.001 and 430 ± 1111 g, *P* = 0.002, respectively) and decreased total fat mass (−495 ± 1464 g, *P* = 0.006 and −351 ± 1403 g, *P *< 0.05, respectively) (Thomas et al., 2021a). Lastly, there were no significant changes following RES or END training in daily caloric intake, active energy expenditure or total energy expenditure (Thomas et al., 2021a), indicating a stable diet and levels of incidental activity (outside of the interventions) throughout the study.

### 3.3 Changes with training: Group means and sex differences

**FIGURE 1:**
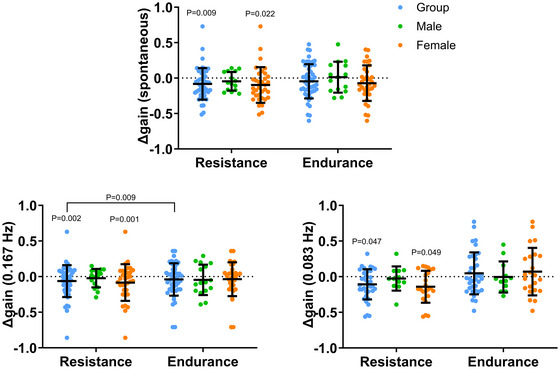
Changes (Δ) in measures of gain during spontaneous dynamic cerebral autoregulation (top) and during forced cerebral dynamic autoregulation at 0.167 Hz (bottom left) and 0.083 Hz (bottom right) following 3 months of END and RES exercise training. Group changes are shown in blue, male changes in green and female changes in orange. The effect of the exercise interventions and the differences between sexes were assessed using a linear mixed model. For changes in spontaneous dynamic cerebral autoregulation gain, *n* = 49 for group, *n* = 15 for male and *n* = 34 for female. For changes in forced dynamic cerebral autoregulation gain at 0.167 Hz, *n* = 36 for group, *n* = 12 for male and *n* = 24 for female. For changes in forced dynamic cerebral autoregulation gain at 0.083 Hz, *n* = 49 for group, *n* = 16 for male and *n* = 33 for female.

**FIGURE 2:**
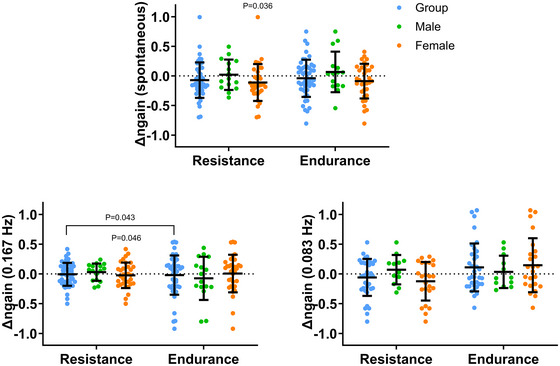
Changes (Δ) in measures of normalized gain (ngain) during spontaneous dynamic autoregulation (top) and during forced dynamic autoregulation at 0.167 Hz (bottom left) and 0.083 Hz (bottom right) following 3 months of END and RES exercise training. Group changes are shown in blue, male changes in green and female changes in orange. The effect of the exercise interventions and the differences between sexes were assessed using a linear mixed model. For changes in spontaneous dynamic cerebral autoregulation ngain, *n* = 49 for group, *n* = 15 for male and *n* = 34 for female. For changes in forced dynamic cerebral autoregulation ngain at 0.167 Hz, *n* = 36 for group, *n* = 12 for male and *n* = 24 for female. For changes in forced dynamic cerebral autoregulation ngain at 0.083 Hz, *n* = 49 for group, *n* = 16 for male and *n* = 33 for female.

**FIGURE 3:**
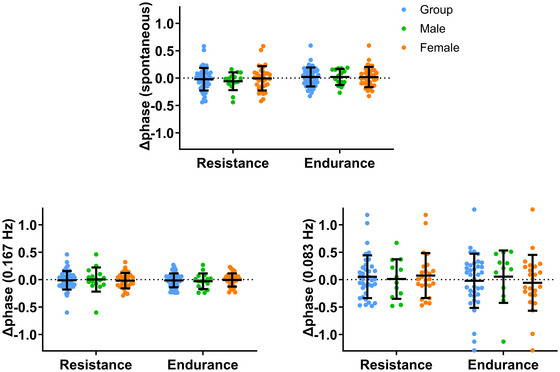
Changes (Δ) in measures of phase during spontaneous dynamic autoregulation (top) and during forced dynamic autoregulation at 0.167 Hz (bottom left) and 0.083 Hz (bottom right) following 3 months of END and RES exercise training. Group changes are shown in blue, male changes in green and female changes in orange. The effect of the exercise interventions and the differences between sexes were assessed using a linear mixed model. For changes in spontaneous dynamic cerebral autoregulation phase, *n* = 49 for group, *n* = 15 for male and *n* = 34 for female. For changes in forced dynamic cerebral autoregulation phase at 0.167 Hz, *n* = 36 for group, *n* = 12 for male and *n* = 24 for female. For changes in forced dynamic cerebral autoregulation phase at 0.083 Hz, *n* = 49 for group, *n* = 16 for male and *n* = 33 for female.

Group mean responses to RES and END training are presented in Table 2 (and Figures 1, 2, 3), and sex‐specific changes in response to training are presented in Table 3 (and Figures 1, 2, 3). The $P_{\mathrm{ET},CO_{2}}$ and blood pressure were unchanged across all dCA assessments (from beginning of manoeuvre to end) and across all four timepoints (before and after each intervention) (all *P *> 0.05).

### 3.4 Changes with training: Group means and sex differences

#### 3.4.1 Spontaneous (BL)

On a group basis, there was a significant decrease in coherence following END training (*P* = 0.003) but not RES training (*P* = 0.558). There was a significant decrease in gain following RES training (*P* = 0.009) but not END training (*P* = 0.175). There were no changes following RES or END training in ngain (RES, *P* = 0.098 and END, *P* = 0.369) or phase (RES, *P* = 0.479 and END, *P* = 0.423).

When male and female responses were separated, there was no change in coherence or phase in either males or females following RES training (males: coherence, *P* = 0.137 and phase, *P* = 0.156; females: coherence, *P* = 0.079 and phase, *P* = 0.907) or END training, except a significant reduction in coherence in females following END training (males: coherence, *P* = 0.100 and phase, *P* = 0.574; females: coherence, *P* = 0.011 and phase, *P* = 0.541). Following END training there were no significant differences in gain or ngain in males (gain, *P* = 0.827 and ngain, *P* = 0.429) or females (gain, *P* = 0.089 and ngain, *P* = 0.078). However, in females there was a significant reduction in gain (*P* = 0.022) and ngain (*P* = 0.036) following RES training, which was not seen in males (*P* = 0.160 and *P* = 0.746, respectively). There was a significant difference between male and female responses following RES but not END in coherence (RES, *P* = 0.026 and END, *P* = 0.987), with a decrease in females and an increase in males.

#### 3.4.2 Forced oscillations (0.167 Hz)

On a group level there was a significant decrease in gain following RES training (*P* = 0.002) but not END training (*P* = 0.330). There were no changes in coherence, ngain or phase following either RES or END training (RES: coherence, *P* = 0.954, ngain, *P* = 0.254 and phase, *P* = 0.400; END: coherence, 0.056, ngain, 0.094 and and phase, 0.808). However, the magnitude of change between RES and END was significantly different for gain (*P* = 0.009) and ngain (*P* = 0.043), with a decrease following RES and increasing following END.

When sex differences were analysed, there was no change following RES training in coherence or phase in either males (*P* = 0.505 and *P* = 0.921, respectively) or females (*P* = 0.502 and 0.354, respectively). However, there was a significant reduction in gain and ngain in females (*P* = 0.001 and *P* = 0.046, respectively) but not males (*P* = 0.581 and *P* = 0.299, respectively) following RES training. There was no difference in coherence, gain, ngain or phase in either males (*P* = 0.076, *P* = 0.932, *P* = 0.890 and *P* = 0.676, respectively) or females (*P* = 0.262, *P* = 0.262, *P* = 0.275 and *P* = 0.573, respectively) following END training.

#### 3.4.3 Forced oscillations (0.083 Hz)

On a group level, there was a significant decrease in gain following RES (*P* = 0.047) but not END (*P* = 0.224). There were no changes in coherence, ngain or phase following either RES (*P* = 0.103, *P* = 0.813 and *P* = 0.610, respectively) or END training (*P* = 0.872, *P* = 0.680 and *P* = 0.450, respectively).

When comparing males and females, females had a significant reduction in gain following RES (*P* = 0.049) and males had a significant reduction in coherence following END (*P* = 0.003). There were no other differences in any variable, in either males or females, after either RES or END training (all *P *> 0.05).

## 4 DISCUSSION

Several lines of evidence suggest that exercise is beneficial for brain health, with impacts on the risk of stroke and dementia (Abbott et al., 1994; Gallanagh et al., 2011; Gill, 2015; Hillman et al., 2008; Lautenschlager et al., 2010; Lautenschlager et al., 2008; Williams, 2009). Despite this, surprisingly little is known regarding the impacts of different forms of exercise training on cerebrovascular physiology, in males and females. In this study, we investigated the hypothesis that different modes of exercise training would impact dCA to a similar extent in both males and females. To this end, we compared 3 months of centre‐based and supervised END and RES training, separated by a 3 month washout. This cross‐over design enabled within‐subject comparison of training modalities that have distinct haemodynamic effects on the cardiovascular system. dCA was assessed via spontaneous and forced blood pressure responses. Our principal findings are as follows: (1) females have reduced dCA (higher gain) and increased MCAv at baseline (when they entered the study) when compared with males; (2) 12 weeks of RES training appears to have a positive impact on both spontaneous dCA (decrease in gain and ngain) and forced dCA (decrease in gain and ngain at 0.167 Hz and gain at 0.083 Hz), whereas END training does not, and this beneficial adaptation following RES training is present in females but not males; and (3) the benefits of RES training are present across multiple methods for measuring dCA (spontaneous and forced dCA at 0.083 and 0.167 Hz). Overall, this investigation into sex differences in both spontaneous and dynamic dCA provides significant insights into the adaptations to acute and rapid changes in blood pressure with different modalities of exercise. Specifically, the addition of dCA quantification using forced oscillations in MAP and MCAv, when compared with our previous work where we studied only spontaneous dCA (Thomas et al., 2021b), provides insight into earlier adaptation, because impairment in dCA may result in slowing of the adaptive response, starting with impairment to counteract rapid blood pressure changes (forced dCA), while the slow changes in blood pressure may remain intact (spontaneous dCA) (Claassen et al., 2021).

dCA is an important regulatory response that stabilizes CBF and nutrient delivery to meet the metabolic demands of the brain. Lower dCA gain has been described as greater damping of the arterial blood pressure drive to CBF (Van Beek et al., 2008), indicating that a reduced dCA gain would be a beneficial adaptation in dCA. We found higher resting gain in females compared with males at baseline, indicating that young females might have worse dCA, which is in line with recent findings by Labrecque et al. (2019), who reported high gain in endurance‐trained females compared with age‐matched males using repeated squat–stands at 0.05 Hz. This could be related to the females potentially having smaller cerebral vessels than their male counterparts (Müller et al., 1991). Considering that we also found reductions in gain and ngain in females following RES training, it is possible that RES evokes beneficial structural adaptations (i.e., changes in vessel diameter) for improving dCA in females, although this might be driven by baseline differences.

As pointed out by Brassard et al. (2023), arguments for using spontaneous as opposed to forced oscillations to assess dCA are ongoing. Current approaches suggest that both techniques have merit. Given that conflicting outcomes in the literature are often explained as a consequence of the use of different dCA measurement techniques, we incorporated both assessments of dCA in order to have a comprehensive assessment. We found that improvement in dCA in females was present across both measurement techniques, indicating consistency of change with training. Spontaneous oscillations used to assess cerebral autoregulation are physiologically representative of daily living (Tzeng & Panerai, 2018; Zhang et al., 1998), and forced oscillations via squat–stand manoeuvres were used in our study because these optimize the signal‐to‐noise ratio and improve the reproducibility of observations by using a physiologically relevant MAP stimulus (Smirl et al., 2015). We found that absolute decreases in transfer function analysis gain and ngain appear greater in the forced dCA at 0.167 Hz and during spontaneous dCA, when compared with forced dCA at 0.083 Hz, suggesting that both methods of dCA assessment are valid in this population. Collectively, the consistency of our forced dCA and spontaneous dCA responses to RES suggests that our findings represent a robust cerebral autoregulation adaptation. Further studies exploring whether these differences are in response to structural changes are warranted.

Changes in transfer function analysis gain and ngain alongside no improvement in transfer function analysis phase could suggest that the mechanisms of dCA improvement are related to vascular compliance. Decreases in gain indicate an improvement in the damping ability of the cerebral circulation, or an improved amplitude modulation, that works to protect the brain from acute insult attributable to large fluctuations in blood pressure. Increased MCA pulsatility with concurrent reduced central arterial compliance in resistance‐trained individuals has been reported in cross‐sectional research (Nakamura & Muraoka, 2018). Furthermore, Zhang et al. (2009) reported that Windkessel models of the cerebral circulation indicate that reductions in cerebral vessel compliance reduce transfer function analysis‐derived gain, indicating that compliance is a modulator of cerebral autoregulation. This effect of compliance was found to predominate in the low‐frequency domain (0.07–0.20 Hz) (Zhang et al., 2009), which is where both forced oscillations fall in the present study. This theory is supported by previous research showing that the myogenic pathway is a primary mechanism underlying pressure‐dependent vasoconstriction and vasodilatation during autoregulation and is thought to make the greatest contribution to cerebrovascular responses to rapid changes in perfusion pressure and flow in the low‐frequency domain (Hamner & Tan, 2014). This suggests that alterations in vascular compliance would have a significant impact on transfer function analysis gain within this frequency (Zhang et al., 1998) and that altered cerebrovascular compliance might underlie the reduced gain and ngain in females following RES training in the present study.

### 4.1 Sex differences

In 2011, Deegan et al. (2011) investigated the difference between the autoregulatory capacity in older men and women (236 males and 308 females) using transfer function analysis gain, phase and coherence. They found that female subjects demonstrated better cerebral autoregulation when compared with males, as evidenced by having lower resting gain and higher phase values. This is in contrast to the present study, in which we found that, at rest, females had poorer cerebral autoregulation (higher resting gain) when compared with males. Our findings are, however, similar to the observations of Wang et al. (2005), who measured transfer function gain in 10 young males and females and reported that women had higher resting gain than men. Given that cerebral autoregulation is an intrinsic mechanism that maintains CBF relatively constant over a wide range of blood pressures, if autoregulatory responses are slow and modest in amplitude, it is possible that transient decreases in blood pressure during postural changes might result in transient cerebral hypoperfusion, potentially causing symptoms including dizziness, light‐headedness and syncope. Interestingly, orthostatic intolerance is three to four times higher in young women than young men (Schondorf et al., 2001). This is supported by Abidi et al. (2017), who reported that women had a greater vasodilatory capacity then men in response to the Valsalva manoeuvre. The improvement in gain and ngain following RES training in females could be attributable to the reduced dCA at baseline. If dCA was poor at baseline, females might have had more potential for improvements in their autoregulatory capacity.

In the present study, females had a higher resting MCAv at baseline when compared with males, which is a consistent finding across the literature (Alwatban et al., 2021). The difference in baseline MCAv between sexes could be explained by differences in MCA vessel diameter and density. Smaller MCA diameter would be likely to result in increased MCAv in female subjects and higher resistance through these vessels, assuming no difference in total CBF. Muller et al. (1991) reported that MCA vessel diameter was 9.3% larger in male subjects. Furthermore, Mouches et al. (2021) collected data on 1722 healthy adults and reported that females showed significantly higher artery density, whereas males showed higher artery radius. In contrast, Orlandini et al. (1985) reported no significant sex differences in cerebral vessels. MCA vessel diameter, flow and density were not measured in the present study; therefore, we cannot exclude the possibility that there were differences in vessel diameter, hence flow. Future studies should consider vessel size and its potential contribution to cerebrovascular resistance and dCA outcomes.

It is possible that female sex hormone levels might have affected the autoregulatory response. In the present study, female menstrual cycle and contraceptive use were not controlled. Favre and Serrador (2019) measured autoregulation in the early follicular, late follicular and midluteal phases during spontaneous and induced blood pressure oscillations in 13 young, healthy women. They concluded that cerebral autoregulation was unchanged across the menstrual cycle. Korad et al. (2022) examined cerebral autoregulation using transfer function analysis across the menstrual cycle in eumenorrhoeic women (*n* = 12; mean ± SD age, 31 ± 7 years) and reported that resting mean MCAv, blood pressure and cerebral autoregulation were unchanged across the menstrual cycle. Furthermore, Abidi et al. (2017) reported no statistically significant evidence for an effect of menstrual cycle or oral contraceptive use on cerebrovascular responses during assessment of postural change. However, they did report a significant impact of oral contraceptive use on MAP (reduction), suggesting a baseline difference in cerebrovascular resistance, which might contribute to the previously mentioned orthostatic intolerance. Together, these studies suggest that acute, normal physiological fluctuations in sex hormones do not substantively impact the ability to maintain CBF during changes in blood pressure, indicating that it is unlikely that oestrogen levels influenced the autoregulatory response in the present study.

### 4.2 Impacts of training modality

Physiological adaptations to exercise are mode specific, with RES exercise increasing muscular strength and mass (Kraemer et al., 1988) and with END exercise exerting more influence on the cardiovascular variables and blood volume and, ultimately, maximal oxygen consumption (Tomoto et al., 2015). These alterations are driven, in part, by the acute haemodynamic impacts of distinct exercise bouts. There remains a paucity of evidence on the impact of RES and END training on cerebrovascular adaptations. However, the present data indicate that RES training provides some beneficial adaptation to dCA in females. Improvements in dCA at a young age could result in beneficial cerebrovascular health with ageing, including reduced microvascular damage and the known increased risk of stroke in postmenopausal women (Intharakham et al., 2019).

Several cross‐sectional studies have investigated dCA in endurance‐trained individuals and have reported increased gain compared with healthy non‐endurance‐trained control subjects or even sedentary individuals. This reduced dCA in endurance‐trained individuals has also been shown via links between increased cardiorespiratory fitness and increased gain (Labrecque et al., 2017). However, in the present study we saw no change in cardiorespiratory fitness with RES training, in the presence of an improvement in dCA, and we saw no change in dCA following END when there was an increase in cardiorespiratory fitness (Marsh et al., 2020). There is less evidence surrounding the impact of chronic RES training on dCA. High‐intensity RES training has been associated with detrimental increases in arterial stiffness in young healthy individuals, yet this association is not true for moderate‐intensity RES (Miyachi, 2013). Conversely, low‐ to moderate‐intensity RES has been shown to have a positive impact via a decrease in pulse wave velocity (Liu et al., 2023). Perry et al. (2019) compared dCA using forced oscillations in blood pressure induced by repeated squat–stand manoeuvres in 12 endurance‐trained individuals (all male), 12 resistance‐trained individuals (all male) and 12 healthy sedentary individuals (mix of males and females). They found no differences in gain or ngain between the groups. Drapeau et al. (2019) also reported a slight attenuation of dCA following 6 weeks of high‐intensity interval training on dCA in endurance‐trained men. However, Perry et al. (2019) and Drapeau et al. (2019) used only men in their exercise groups, whereas we saw changes only in females, which might explain why we saw differences in our data set and why Perry et al. (2019) and Drapeau et al. (2019) reported no impact of training. Furthermore, Perry et al. (2019) used habitual training rather than an exercise intervention, which might result in a misinterpretation of exercise results.

It is likely that the cerebrovascular responses observed in the present investigation stem from a complex interplay of countervailing adaptations within central and peripheral circulations that are exercise modality specific. RES training might result in more frequent and larger fluctuations in blood pressure. These adaptations observed in the RES group might alter dCA more than END training. Furthermore, dCA is more sensitive to reductions in MAP (hypotension) than increases in MAP (Tzeng et al., 2010). During RES training, an individual will experience frequent episodes of hypertension during a lift or contraction, with subsequent relative hypotension after a lift. As a result, frequent oscillations are likely to occur in vasomotion of the cerebrovasculature. Our findings suggest that the interplay between hyper‐ and hypotension experienced during RES training might provoke beneficial dCA adaptations. These bouts of hypo‐ and hypertension are perhaps less apparent during END training. Data documenting the directionality of the cerebral pressure–flow relationship in RES and END training are significantly lacking. However, Roy et al. (2022) investigated this directional sensitivity in 12 RES‐trained and 12 END‐trained men and reported that a pattern of directional sensitivity of the cerebral pressure–flow relationship was present in END‐trained individuals but absent in RES‐trained individuals. However, these findings are based on habitually trained individuals and cannot be compared directly with results from a supervised training intervention, nor can they be extrapolated to responses that might be seen in females. Comparing directional sensitivity analysis in males and females following different modalities of exercise would be a valuable contribution for future research.

### 4.3 Limitations

This study had several limitations. Firstly, the use of transcranial Doppler to assess CBF in the MCA relies on the assumption that the diameter of these vessels does not change during cerebrovascular assessments or in response to the training interventions. Although structural remodelling of the cerebrovasculature has not been confirmed, there is evidence suggesting that exercise remodels peripheral blood vessels in humans (Green, 2009; Green et al., 2010, 2017; Joyner & Green, 2009; Naylor et al., 2011; Tinken et al., 2010). Nonetheless, we did not see remodelling of the internal carotid artery after either exercise intervention in a similar group of subjects (Thomas et al., 2021b, 2022). Secondly, this study was limited to the assessment of END and RES and did not address the influence of other modalities (e.g., high‐intensity interval training or combined RES and END). Thirdly, owing to the constraints of testing time points in the present study, we did not control for menstrual cycle phase. This might have had implications for the reported changes with training and baseline differences seen between males and females (Abidi et al., 2017). Fourthly, previously established guidelines suggest a minimum of 5 min of recording of spontaneous fluctuations of arterial blood pressure and CBF for transfer function analysis (Panerai et al., 2023). The present study did use a 5 min recording for the spontaneous dCA analysis; however, only a 3 min recording was used for the forced dCA analysis. This might have resulted in a decrease in data quality and increase in excluded data points in the forced dCA data. However, recording of 3 min can be acceptable in protocols that induce dynamic changes in blood pressure, as done with repeated squat–stands in the present study (Panerai et al., 2023). Finally, although research surrounding young healthy populations is crucial in determining mechanisms surrounding cerebrovascular adaptation to exercise training, we cannot extrapolate our findings to older populations or to patients with impaired cerebrovascular function, such as reduced cognitive function, stroke or dementia.

## 5 CONCLUSION

This study is the first to assess sex differences in dCA using a randomized cross‐over within‐subject experimental design involving two distinct exercise modalities. These modalities, and the programmes we designed, are ecologically valid in that they emulate guideline‐based (Haskell et al., 2007; Nelson et al., 2007) programmes similar to those typically and widely adopted in the community to enhance fitness or muscular strength and hypertrophy. Our findings suggest that females are at a greater risk of poor dCA owing to having lower transfer function gain at rest prior to commencement of training. However, our results suggest that females have greater potential for improvements in dCA with exercise in comparison to males, specifically with RES training. Of note, this study was the first to compare the impact of RES and END training on forced dCA, in contrast to cross‐sectional comparisons of trained individuals. Future research should aim to assess the impact of interventions rather than training status to gain a better understanding of the impacts on dCA. Furthermore, future training studies should continue to use multiple methods of measuring dCA to increase comprehensive (i.e., rigour and confidence) reporting in experimental findings.

dCA has been shown to be impaired in numerous cerebrovascular diseases, such as stroke and dementia, with the risk of stroke and dementia postmenopause being higher in females (Intharakham et al., 2019). We have shown that dCA can be improved in young women following RES training. Incorporating supervised and individualized RES training into preventative exercise programmes for individuals at risk of cerebrovascular disease could be beneficial in improving blood pressure control and regulation of CBF. However, we acknowledge that adaptation in young, healthy individuals might not be generalizable to older or clinical populations. Nonetheless, establishing baseline sex‐specific exercise responses in healthy individuals before exploring more complex clinical populations is important. Future research should investigate both the impact of different exercise interventions on clinical populations and the impact of longer interventions to determine whether RES training can have benefits on cerebrovascular adaptation in males or whether END training can have benefits or detriments on dCA in either sex.

## AUTHOR CONTRIBUTIONS

Experiments in this study were performed at the University of Western Australia, School of Human Sciences laboratory. Daniel J. Green, Louise H. Naylor and Kurt J. Smith conceived, designed, funded and supervised the research. Hannah J. Thomas, Channa E. Marsh and Kurt J. Smith collected the data. Hannah J. Thomas, Daniel J. Green, Kurt J. Smith, and Leanne Lester analysed and presented the data. Hannah J. Thomas, Daniel J. Green and Kurt J. Smith prepared the figures. Hannah J. Thomas, Kurt J. Smith, Louise H. Naylor and Daniel J. Green interpreted the results of the experiment and drafted the manuscript. All authors critically revised and approved the final version of manuscript and agree to be accountable for all aspects of the work in ensuring that questions related to the accuracy or integrity of any part of the work are appropriately investigated and resolved. All persons designated as authors qualify for authorship, and all those who qualify for authorship are listed.

## CONFLICT OF INTEREST

The authors declare that they have no conflicts of interest.

## Data Availability

All original ‘raw’ data are available upon reasonable request.
